# Central Canal Ependymal Cells Proliferate Extensively in Response to Traumatic Spinal Cord Injury but Not Demyelinating Lesions

**DOI:** 10.1371/journal.pone.0085916

**Published:** 2014-01-27

**Authors:** Steve Lacroix, Laura K. Hamilton, Alexandre Vaugeois, Stéfanny Beaudoin, Christian Breault-Dugas, Isabelle Pineau, Sébastien A. Lévesque, Catherine-Alexandra Grégoire, Karl J. L. Fernandes

**Affiliations:** 1 Department of Neurosciences, Faculty of Medicine, Centre de recherche du Centre hospitalier de l’Université de Montréal (CRCHUM), and Groupe de recherche sur le système nerveux central (GRSNC), Université de Montréal, Quebec, Canada; 2 Centre de recherche du Centre hospitalier universitaire (CHU) de Québec – CHUL et Département de médicine moléculaire, Faculté de médecine, Université Laval, Québec, Canada; Heidelberg University Hospital, Germany

## Abstract

The adult mammalian spinal cord has limited regenerative capacity in settings such as spinal cord injury (SCI) and multiple sclerosis (MS). Recent studies have revealed that ependymal cells lining the central canal possess latent neural stem cell potential, undergoing proliferation and multi-lineage differentiation following experimental SCI. To determine whether reactive ependymal cells are a realistic endogenous cell population to target in order to promote spinal cord repair, we assessed the spatiotemporal dynamics of ependymal cell proliferation for up to 35 days in three models of spinal pathologies: contusion SCI using the Infinite Horizon impactor, focal demyelination by intraspinal injection of lysophosphatidylcholine (LPC), and autoimmune-mediated multi-focal demyelination using the active experimental autoimmune encephalomyelitis (EAE) model of MS. Contusion SCI at the T9–10 thoracic level stimulated a robust, long-lasting and long-distance wave of ependymal proliferation that peaked at 3 days in the lesion segment, 14 days in the rostral segment, and was still detectable at the cervical level, where it peaked at 21 days. This proliferative wave was suppressed distal to the contusion. Unlike SCI, neither chemical- nor autoimmune-mediated demyelination triggered ependymal cell proliferation at any time point, despite the occurrence of demyelination (LPC and EAE), remyelination (LPC) and significant locomotor defects (EAE). Thus, traumatic SCI induces widespread and enduring activation of reactive ependymal cells, identifying them as a robust cell population to target for therapeutic manipulation after contusion; conversely, neither demyelination, remyelination nor autoimmunity appears sufficient to trigger proliferation of quiescent ependymal cells in models of MS-like demyelinating diseases.

## Introduction

Ependymal cells (EpCs) of the adult spinal cord are an enigmatic population of ciliated cells lining the central canal. They are comprised of multiple morphologically identifiable subtypes, including cuboidal EpCs and radial tanycytic EpCs [1]–[5], and are descended developmentally from embryonic neuroepithelial stem cells located in the ventral neural tube [6]–[8]. In lower vertebrates, such as certain types of fish, amphibians and reptiles, EpCs play a central role in the remarkable regeneration observed following spinal cord transection [9]–[11]. However, in the adult mammalian spinal cord, which is notable for its lack of regenerative ability, EpCs are thought to primarily serve endocrinological and protective roles [4], and exhibit relatively low basal mitotic activity [2], [12]–[14].

Despite the limited anatomical and functional regeneration observed following damage to the adult mammalian spinal cord [15], [16], recent studies indicate that reactive mammalian EpCs re-express neural stem cell properties following spinal cord injury (SCI) [1], [17], [18]. Mechanical damage to the central canal triggers a robust increase in EpC proliferation [12], [19], [20], and an upregulation in expression of the neuroepithelial stem cell marker Nestin [19], [21]. This is followed by migration of EpC-derived progeny to the site of injury [12], [20], where they exhibit multi-lineage differentiation into both astrocytes and oligodendrocytes [1], [17], [22]. Consistent with a latent neural stem cell potential, tissue culture and fate-mapping experiments have revealed that virtually all stem-like cells that can be isolated from the intact or injured spinal cord using the colony-forming neurosphere assay are derived from the EpC population [17].

The induction of stem cell properties in reactive EpCs raises the fascinating possibility of targeting these endogenous cells to promote spinal cord repair. For example, reactive EpCs could be targeted to modulate their astrocytic contribution to the glial scar; to increase the size of the oligodendrocyte progenitor pool; or to potentially generate interneurons capable of relaying impulses across the injury site. However, important questions remain unanswered, including the timing, location and extent of EpC reactions to an injury, and the types of damage to spinal cord tissue that are capable of inducing EpC reactions. In the present study, we have investigated in detail the pattern of EpC activation in response to spinal cord pathologies. We first examined the spatiotemporal dynamics of injury-induced EpC proliferation using the Infinite Horizon (IH) impactor, a clinically relevant model of spinal cord contusion injury [23]. We then asked whether non-traumatic spinal cord pathologies are capable of eliciting EpC activation, focusing on the lysophosphatidylcholine (LPC, also called lysolecithin) model of focal demyelination and the experimental autoimmune encephalomyelitis (EAE) model of multi-focal demyelination. Our findings provide insights relevant to the development of potential therapeutic strategies for using endogenous EpCs to promote repair of the injured or diseased spinal cord.

## Materials and Methods

### Animals

A total of 165 adult female C57BL/6 mice were used in this study. Mice were purchased from Charles River Laboratories (St-Constant, QC, Canada) and had free access to food and water. All surgical procedures were approved by the Laval University Animal Care Committee and followed Canadian Council on Animal Care guidelines. The distribution of mice used among the three different models of spinal pathologies and time points at which animals were sacrificed are reported in Table 1.

**Table 1:** Overview of the number of mice included in experiments.

Group	Time of sacrifice (days)						
	0	1	3	7	14	21	35
***SCI (n = 50)***							
1. Naive	4						
2. Sham		4				4	
3. SCI		10	6	6	6	5	5
***LPC (n = 92)***							
1. PBS-injected			4	12	4	9	8
2. LPC-injected			9	19	9	9	9
***EAE (n = 23)***							
1. Naive	5						
2. Immunized				5	4	5	4

### Spinal Cord Injury (SCI)

C57BL/6 (N  = 46) mice were anesthetized with isoflurane and underwent a laminectomy at vertebral level T9–10, which corresponds to spinal segment T10–11. Briefly, the vertebral column was stabilized and a contusion of 50 kdyn (moderate injury) was performed using the IH SCI device (Precision Systems & Instrumentation, Lexington, KY), typically resulting in a spinal cord displacement of 400 to 500 µm. Overlying muscular layers were then sutured and cutaneous layers stapled. Post-operatively, animals received manual bladder evacuation twice daily to prevent urinary tract infections. SCI mice were sacrificed by perfusion at 1, 3, 7, 14, 21, and 35 days post-contusion. Naive (N = 4) and sham-operated mice were used as controls.

### Lysophosphatidylcholine (LPC)-induced Demyelination

A laminectomy was done to expose the spinal cord at vertebral level T9–10, and 1 µl of either L-α-LPC (L-α-lysolecithin, catalog # L-1381; Sigma-Aldrich Canada, Oakville, ON, Canada) at a concentration of 1 µg/µl or PBS injected into the dorsal column or dorsolateral funiculus (DLF) white matter using a glass micropipette with a tip diameter of 40 µm, as previously described [24]. Mice (N  = 92) were sacrificed at 3, 7, 14, 21, and 35 days post-injection.

### Experimental Autoimmune Encephalomyelitis (EAE)

Induction of EAE in C57BL/6 mice (N  = 18) was used as a model for multiple sclerosis (MS). EAE was induced by subcutaneous injections of 100 µg of myelin oligodendrocyte glycoprotein (MOG_35–55_; MEVGWYRSPFSRVVHLYRNGK, AnaSpec Inc., Fremont, CA) in complete Freund’s adjuvant (incomplete Freund’s adjuvant containing 4 mg/ml of heat-inactivated *Mycobacterium tuberculosis* H37Ra; BD Biosciences, Mississauga, ON, Canada). An intravenous injection of 200 ng of pertussis toxin (PTX; List Biological Laboratories Inc., Campbell, CA) was also administered on days 0 and 2 of the immunization. The severity of EAE was scored daily using a grading scale of 0–5, following recommendations of Stromnes and Goverman [25]: 0 =  unaffected, 0.5 =  partially limp tail, 1 =  paralyzed tail, 2 =  hind limb paresis and loss in coordinated movement, 2.5 =  one hind limb paralyzed, 3 =  both hind limbs paralyzed, 3.5 =  hind limbs paralyzed and weakness in forelimbs, 4 =  forelimbs paralyzed, and 5 =  moribund/death. Animals were sacrificed at 7, 14, 21, and 35 days post-immunization. Naive mice (N = 5) were used as controls.

### Tissue Processing and Histology

Spinal cords were collected and prepared as previously described [26], [27]. Briefly, mice were overdosed with a mixture of ketamine-xylazine and transcardially perfused with 4% paraformaldehyde (PFA), pH 7.4, in PBS. Spinal cords were dissected out, post-fixed for 2 days at 4°C, and then transferred for 2 days into PBS containing 30% sucrose. For each SCI mouse, a spinal cord segment of 12 mm centered over the lesion site was cut in 12 series of 30-µm-thick coronal sections using a cryostat. In addition, a 4-mm spinal segment corresponding to the C4–6 level was isolated and processed for each SCI animal. Cervical, thoracic and lumbar spinal segments corresponding to the same levels, respectively C4–6, T5–12 and L1–4 levels, were isolated from the spinal cord of LPC-injected and EAE mice as well as control animals. All sections were collected directly onto Surgipath X-tra microslides having a permanent positive charged surface (Leica Microsystems Canada, Concord, ON, Canada) and stored at −20°C until use. To identify the site of SCI and visualize demyelinating lesions, one series of adjacent sections was stained with cresyl violet (CV) and/or Luxol Fast blue (LFB).

### Immunohistochemistry

Primary antibodies used in this study were mouse anti-human Ki67 (1∶200, BD Biosciences), chicken anti-Vimentin polyclonal (1∶1000, Millipore, Billerica, MA), rabbit anti-mouse Olig2 monoclonal (1∶250, Millipore). Brightfield immunohistochemistry for Ki67 was performed using a biotinylated anti-mouse secondary antibody (Jackson ImmunoResearch, West Grove, PA) in conjunction an avidin-biotin-peroxidase amplification system (VectaStain ABC Kit, Vector Laboratories, Burlington, ON, Canada) and 3,3-diaminobenzidine (DAB). Brightfield sections were counterstained with CV. Multilabel immunofluorescence was performed by co-incubating cryostat sections with Ki67, Olig2 and Vimentin primary antibodies and detecting them with Alexa 555 goat anti-mouse (1∶1000), Alexa 488 goat anti-rabbit (1∶1000) and Dylight donkey anti-chicken (1∶500) secondary antibodies (Jackson ImmunoResearch). Immunofluorescence sections were counterstained with Hoechst 33342 (0.2 µM, Sigma-Aldrich Canada).

### Microscopy

Brightfield images were captured using an Olympus BX43F microscope equipped with an Olympus DP-21 color camera. Immunofluorescence images were captured using an Olympus IX81 motorized microscope equipped with a Retiga 2000RV black and white CCD camera (Q-Imaging, Surrey, BC, Canada) and a Metamorph image acquisition/analysis software (Molecular Devices Corporation, Sunnyvale, CA). Co-localization of immunofluorescence signals were confirmed using Z-stacks, which were subsequently processed using a 2D deconvolution function and flattened by Maximum projection for presentation. Images were colorized and merged using Adobe Photoshop (v. CS5). Figures were assembled using Adobe Illustrator (v. CS5).

### Quantification and Statistical Analysis

Counts of Ki67+ cells within the central canal ependymal zone were made using a 40x objective at a total magnification of 400x. Ki67+ cells were quantified from each of the four tissue blocks (cervical, rostral, medial, caudal) of every animal. Proliferation as a function of distance from the lesion epicenter was assessed by 7 cryostat sections located at 500–600-µm intervals in each block. Mean Ki67+ cells/section in each tissue block were obtained by averaging the cell counts from all sections of a block.

Statistical analyses of Ki67 cell counts were made using the Graph-Pad Prism software for Windows, version 5.0a (GraphPad Software Inc., San Diego, CA). In the SCI model, differences between the group means were tested by 1-way ANOVA followed by comparison against the Sham group using Dunnett’s post-hoc test. In the LPC model, LPC and PBS conditions were compared against each other at each time point by t-test. In the EAE model, differences between group means were compared by 1-way ANOVA. A *p* value <0.05 was considered as statistically significant. Error bars represent the standard error of the mean (SEM) of each group.

## Results

### Experimental Animal Models and Design

**Figure 1:**
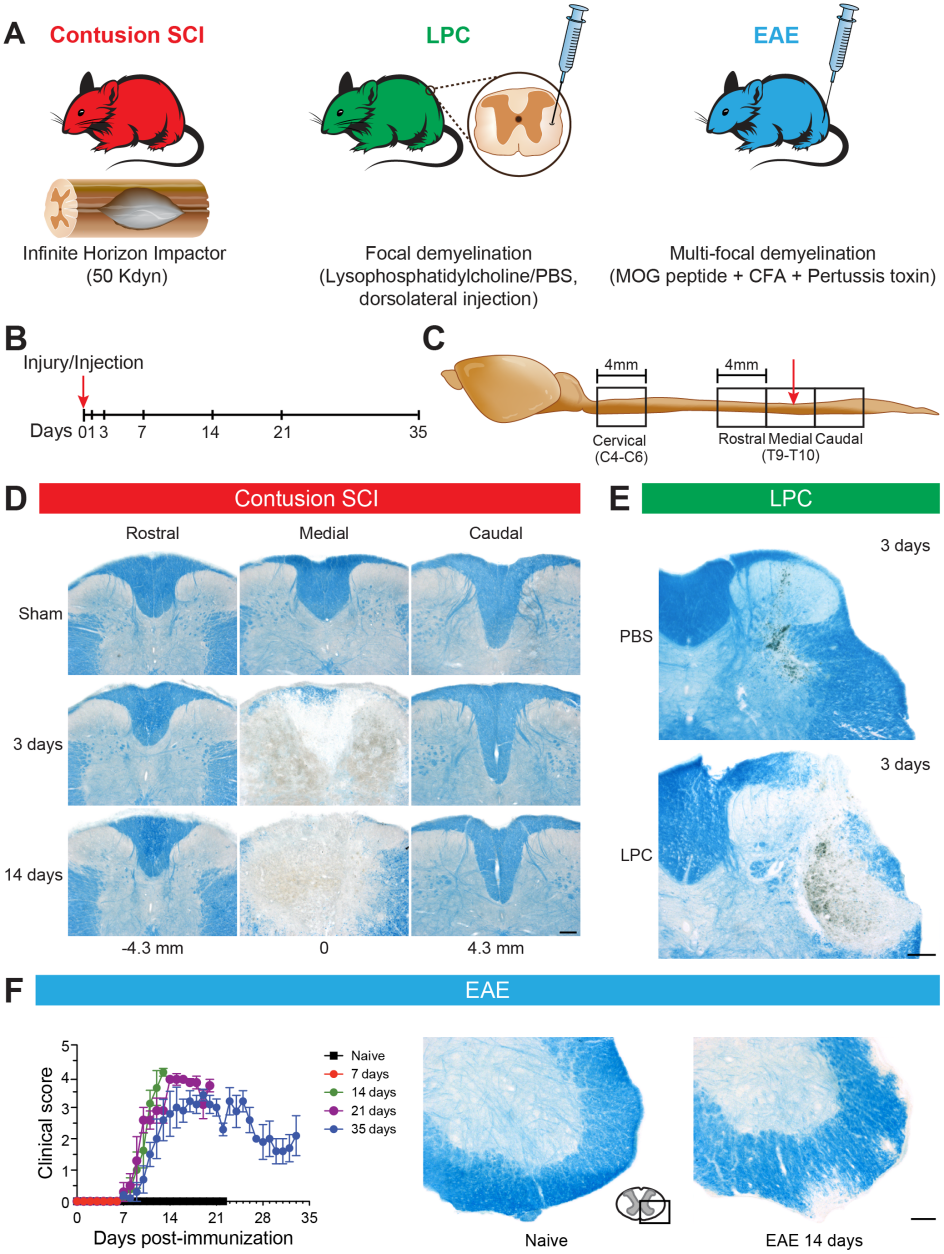
Description of SCI, LPC and EAE models of spinal cord pathologies. (**A**) Models of spinal cord pathologies. (**B**) Time course of mouse sacrifices. Day 0 is the time of contusion SCI, intraspinal injection of lysophosphatidylcholine (LPC) or PBS, or peripheral injection of EAE-inducing MOG/CFA/Pertussis toxin. Only SCI and LPC mice were sacrificed at days 1 and 3, due to the slow onset of EAE following immunization. (**C**) Spinal cord segments harvested for sectioning and analysis. The red arrow points to the lesion epicenter. (**D**) Gross histology of the low thoracic spinal cord following contusion SCI. Shown are representative examples of Luxol Fast blue (LFB)-stained sections taken from the Rostral segment (left column), Medial segment (middle column) and Caudal segment (right column) of a sham-operated mouse (sacrificed at day 21) and SCI mice at 3 and 14 days post-injury. Approximate distances (in millimeters) are shown from the lesion epicenter at the middle of the Medial segment (0). (**E**) Gross histology of the T9–10 thoracic spinal cord injection of PBS (upper panel) or LPC (lower panel) in the dorsolateral funiculus. Luxol Fast blue stains the white matter myelin, and reveals the large zone of demyelination already evident at 3 days after LPC injection. (**F**) Clinical scores and histopathology of EAE mice. Note the rapid increase in clinical scores between 1 and 21 days after immunization, reflecting declining locomotor function that stabilizes and slightly improves between 21 and 35 days after immunization (left). See Methods for description of clinical scores. Luxol Fast blue staining documented the presence of demyelinating plaques spread in the spinal cord white matter of EAE mice at 14 days post-immunization (right). Scale bars: **D**–**F** 100 µm.

Previous studies have shown that proliferation of central canal EpCs is induced in various rodent models of SCI. To better understand EpC reactions to different forms of tissue injury, we compared their responses in three distinct models of traumatic and non-traumatic spinal cord pathologies (Fig. 1A). As a clinically-relevant model of traumatic SCI, we used the IH impactor model of spinal cord contusion, which generates highly reproducible lesions that recapitulate key pathological features of human SCI [27], [28]. A medium-impact 50 kdyn IH force was used in order to preserve as much as possible the cytoarchitecture of the ependymal layer and limit motor deficits. In parallel, we used two non-traumatic models of MS-type demyelination. To create focal demyelination, we injected LPC into spinal cord white matter. Following intraspinal injection of LPC, myelin breakdown is rapidly induced in a restricted zone and damaged myelin sheaths are phagocytosed by immune cells within only a few days [24], [29]. Importantly, demyelination occurs in the LPC model while axons and neighboring cells remain relatively intact, thus allowing for remyelination [30]. As an MS-like model of autoimmune-mediated, multi-focal demyelination, we performed peripheral injections of MOG_35–55_ emulsified in complete Freund’s adjuvant and Pertussis toxin to generate EAE. MOG-induced EAE is a slow onset, autoimmune-mediated process that results in widespread CNS demyelinating lesions and development of quantifiable locomotor deficits [25]. SCI, LPC, and EAE mice were sacrificed at intervals up to 35 days post-spinal cord contusion/intraspinal injection/peripheral immunization (Fig. 1B). Following perfusion fixation, a 12-mm long segment encompassing the mid thoracic and upper lumbar regions of the spinal cord was removed (in the case of SCI and LPC models, centered on the T9/10 contusion or LPC/PBS injection site) and divided into three 4 mm segments (Rostral, Medial and Caudal) (Fig. 1C). In addition, a 4-mm long segment of the mid-cervical (Cervical) spinal cord was harvested for examination of possible changes distant to the injury site (Fig. 1C).

### Histopathology of Contusion SCI, LPC and EAE Models

#### Contusion SCI

Tissue sections at regular intervals across the three thoracic tissue blocks were stained with LFB to examine gross changes in tissue morphology (Fig. 1D). Extensive tissue damage was apparent at the lesion site at 3 days post-injury (dpi), extending ventrally from the dorsal surface and, at the very center of the lesion, often resulting in disappearance of the central canal. Importantly, however, the central canal was preserved both rostral and caudal to the lesion epicenter (even within the Medial block). At 3–14 dpi, edema was particularly pronounced in the caudal direction from the epicenter and was focused within the dorsal column white matter, extending ventrally to the perimeter of the central canal (Fig. 1D). Smaller pockets of blood were intermittently found rostral to the lesion epicenter as well. Interstitial blood was no longer apparent by 14 dpi, and at the lesion epicenter, there was extensive reactive gliosis in the dorsal half of the spinal cord (data not shown). These observations confirmed that the 50 kdyn contusion paradigm would sufficiently spare the central canal to enable further analyses.

#### LPC-induced focal demyelination

To create a focal zone of rapid demyelination in a specific white matter location, separate mice were injected intraspinally with 1 µl of LPC using a fine glass micropipette with an outer diameter of <40 µm. Our initial experiments were focused on injections into the dorsal column, but over-insertion of the micropipette due to breathing movement was sometimes observed to result in traumatic disturbance of the central canal. We thus decided to opt for a model in which injections were performed in the DLF. A representative example of focal demyelination observed at 3 days after injection is shown in Fig. 1E, as visualized using LFB staining. No demyelination was detected in the DLF of PBS-treated animals. Histological observations revealed that demyelination was considerable on days 3, 7, 14 and 21 post-LPC injection and that remyelination was well advanced by 35 days. Importantly, the cytoarchitecture of the central canal was always preserved with the DLF injections.

#### EAE-induced multi-focal demyelination

To generate an immune response leading to a more disseminated pattern of demyelinating lesions in the spinal cord, reminiscent of MS plaques, a third series of mice was immunized with MOG in complete Freund’s adjuvant. Evaluation of the EAE clinical scores was performed on a daily basis and results are reported in Fig. 1F (left panel). On average, disease onset (i.e. appearance of first signs of paralysis) was observed at day 9.2±0.4. At the peak of disease (day 13.5±0.8), the mean clinical score for EAE mice was 4.0±0.1. Histological staining with LFB at day 14 (peak of disease) confirmed the presence of many demyelinating plaques in the spinal cord of EAE mice, but not in control naive mice (Fig. 1F, right panels).

### Gross Proliferative Changes in the Spinal Cord

**Figure 2:**
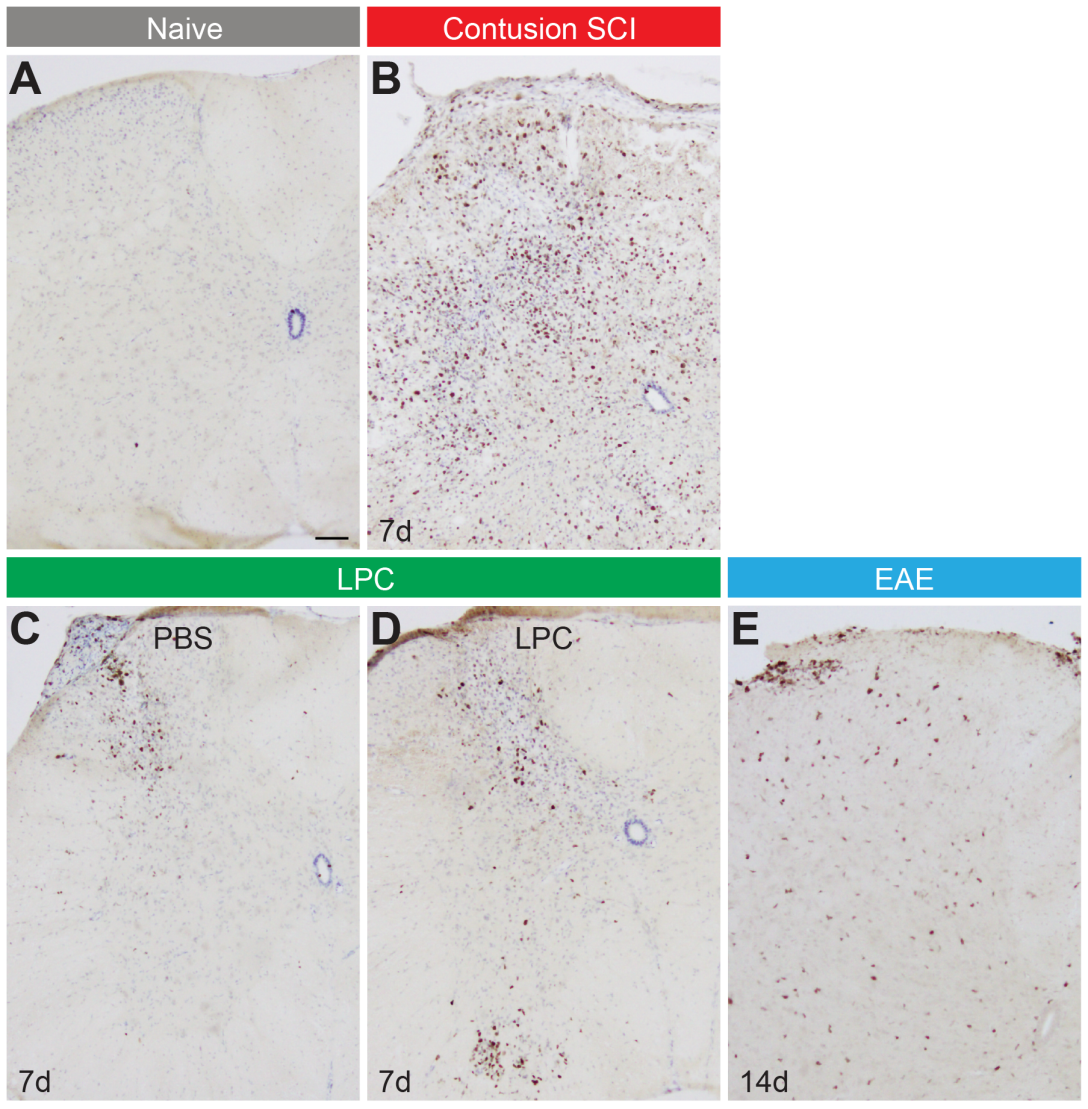
Distinct proliferative reactions in the spinal cord following SCI, LPC and EAE. (**A–E**) Low magnification images of the spinal cord following immunohistochemistry for the cell proliferation marker Ki67 in a naive mouse (**A**), 7 days following contusion SCI (**B**), 7 days following intraspinal injection of PBS (**C**) or LPC (**D**), or 14 days following peripheral immunization in EAE mice (**E**). Scale bar: **A**–**E**, 80 µm.

Distinct macroscopic patterns of proliferative changes were observed in the spinal cords across these three types of pathologies (Fig. 2). Similar to previous studies [2], [14], naive animals possessed only rare cells expressing the proliferation-associated antigen Ki67, and these could be found in the white matter, grey matter and/or ependymal zone (Fig. 2A). Seven days following contusion SCI, large numbers of Ki67+ cells were evident in the lesion segment; these cells were concentrated in, but not limited to, the affected dorsal half of the spinal cord (Fig. 2B). At the same 7 day time-point following intraspinal injection of LPC or PBS control (Fig. 2C–D), Ki67+ cells were likewise increased in the spinal cord compared to non-injected controls (naive); however, these cells were largely restricted to the tissue along the needle tract. At 14 days following EAE induction, corresponding to the approximate peak of locomotor deficits, increased numbers of Ki67+ cells were detectable and were distributed throughout the dorso-ventral extent of the spinal cord (Fig. 2E). Thus, increases in overall cell proliferation occurred in all three models of spinal cord pathology, but with distinct spatial patterns.

### Robust, Long-distance, and Asymmetric Increase in Proliferation in the Ependymal Zone Following Contusion SCI

**Figure 3:**
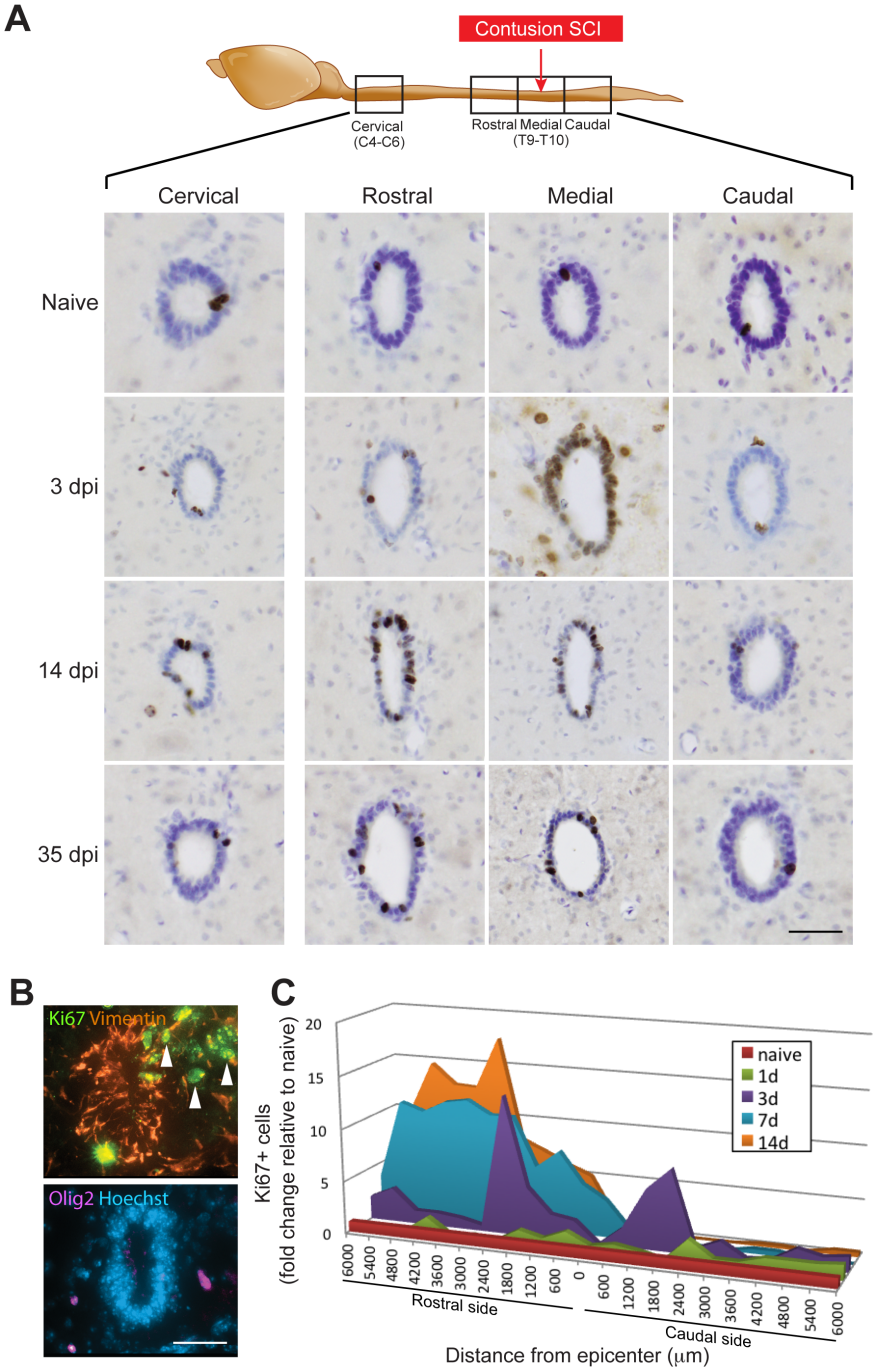
Changes in ependymal zone proliferation following contusion SCI. (**A**) Immunohistochemistry for Ki67-positive (+) proliferating cells in each harvested segment of the spinal cord of naive mice and 3, 14, or 35 days post-injury (dpi). Sections are counterstained with cresyl violet. Note the increased cell proliferation in the ependymal zone of the Medial, Rostral and Cervical spinal cord segments in SCI mice compared to naive animals. (**B**) Multilabel fluorescence immunostaining of the ependymal zone at 7 dpi. Upper panel shows immunostaining for Vimentin (orange) and Ki67 (green) while lower panel shows the same field labeled with Olig2 (fucia) and Hoechst counterstaining (blue). Note that Vimentin+/Ki67+ proliferating EpCs can be seen leaving the central canal ependymal zone (arrowheads). (**C**) Graph of the spatial and temporal progression of changes in ependymal zone proliferation following contusion SCI. Scale bars: **A**, 40 µm; **B**, 35 µm.

We next focused on the proliferative changes occurring in the ependymal zone in the contusion model of SCI (Fig. 3). Naive (non-injured) mice averaged 1–3 proliferating cells/30-µm diameter coronal section in the ependymal zone at cervical, thoracic and lumbar levels of the spinal cord. Following contusion SCI, characteristic patterns of proliferative changes were observed within each tissue block (Fig. 3A). In the lesioned Medial segment, ependymal zone proliferation rapidly increased, peaking at 3 dpi and subsequently declining before peaking again at 14 dpi. Within this Medial segment, the central canal was ablated at the lesion epicenter, but proliferation in the ependymal zone was identifiable within 600 µm rostral and caudal to the injury, and peaked at a distance of approximately 1800 µm (Fig. 3C). In the adjacent Rostral segment, proliferation increased more gradually, peaking at 14 dpi and remaining increased at 21–35 dpi (Fig. 3A). Although not statistically significant, a trend toward an increase in the proliferation of EpCs was observed at greater distances rostral to the lesion site, as a smaller and slower increase in ependymal zone proliferation was detected in the Cervical block (Fig. 3A), which peaked at 21 dpi (not shown). In marked contrast to the Medial, Rostral and Cervical blocks, ependymal zone proliferation in the segment Caudal to the lesion segment did not increase at any time-point following contusion injury.

Fluorescence immunohistochemistry confirmed that Ki67+ cells surrounding the traumatically injured central canal expressed the EpC markers Sox2 (not shown) and Vimentin (Fig. 3B). Ki67+ cells that were weakly Vimentin+ could occasionally be observed migrating away from the central canal after SCI (Fig. 3B), consistent with previous lineage tracing data [1]. Olig2+ oligodendrocyte progenitors, the principle proliferating cell type in the non-injured spinal cord, were not found to express Ki67+ surrounding the injured central canal (Fig. 3B).

### Limited Ependymal Responses in LPC and EAE Models of Demyelination

**Figure 4:**
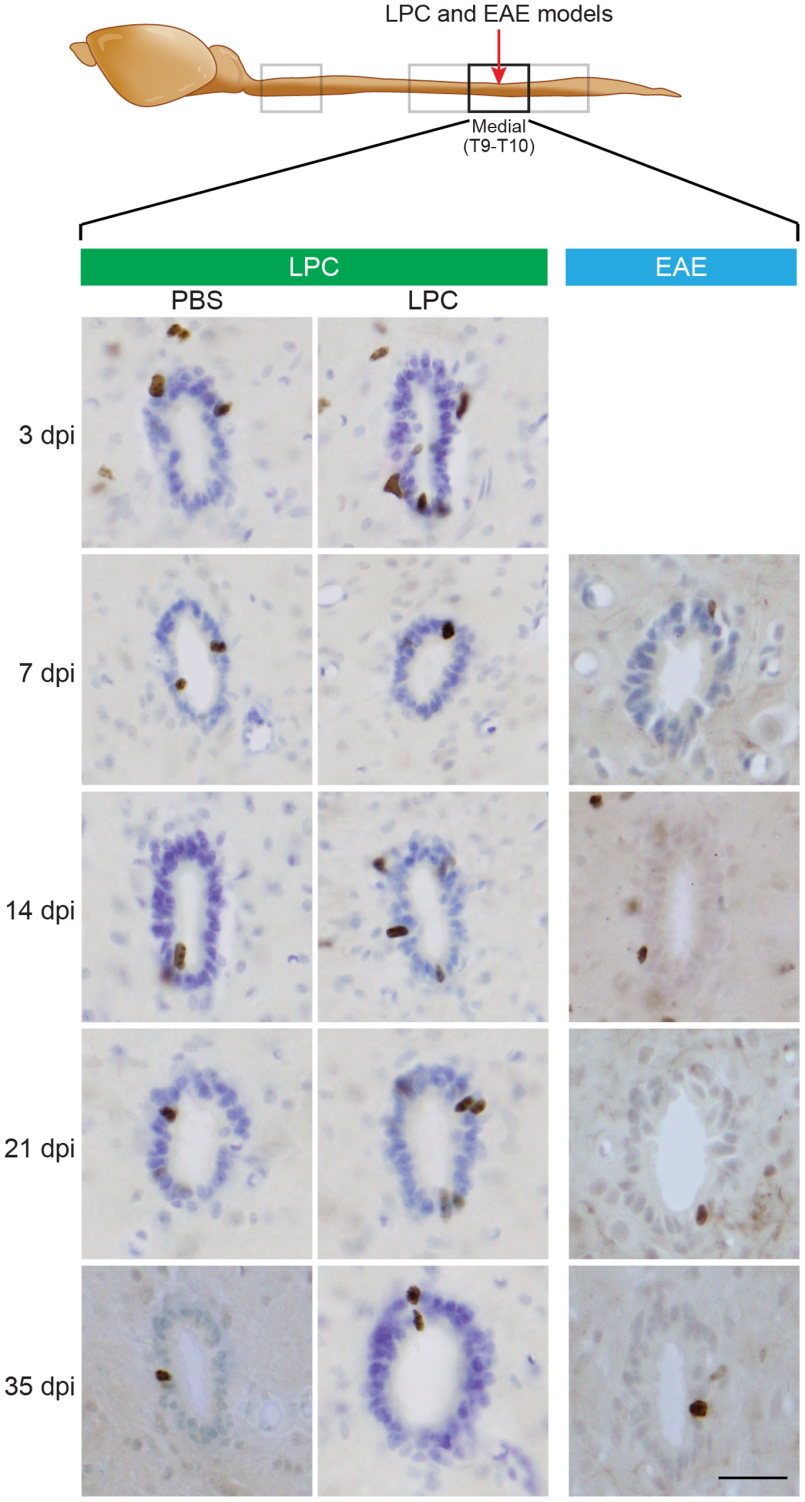
Ependymal cells do not proliferate in response to LPC and EAE models of demyelination. Immunohistochemistry for Ki67-positive (+) ependymal zone cells in the Medial segment of mice injected intraspinally with PBS (left column) or lysophosphatidylcholine (LPC, middle column), or injected peripherally to induce EAE (right column). Images are from mice sacrificed 3, 7, 14, 21, or 35 days post injection/immunization (dpi). Scale bar  = 35 µm.

**Figure 5:**
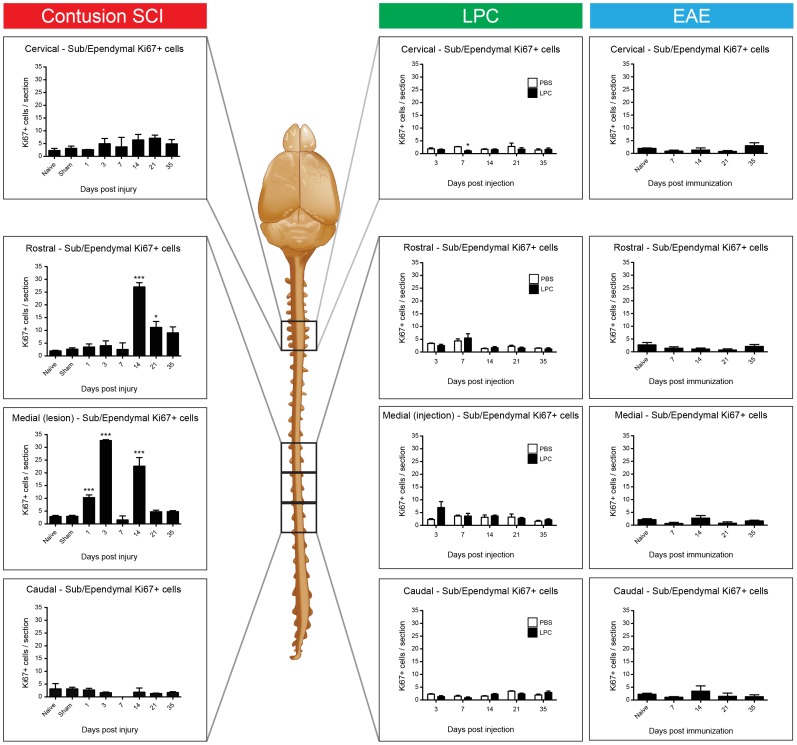
Comparison of spatiotemporal changes in ependymal proliferation after contusion SCI, intraspinal LPC injection or MOG immunization to induce EAE. Complete summary of Ki67+ cell quantifications from the Cervical and three thoracic (Rostral, Medial, Caudal) segments of the spinal cord in all three models of spinal cord pathologies.

In the LPC model of focal demyelination, ependymal zone proliferation was assessed using time point-matched injections of LPC versus PBS into the dorsal columns (Fig. 4). In contrast to SCI, there was no detectable LPC-induced increase in ependymal Ki67+ cells (compared to PBS) in any segment or at any time point examined. While quantification showed that PBS injection did by itself cause a small baseline increase in ependymal zone proliferation within the Medial and Rostral segments (Fig. 5), this was attributable to a localized needle-induced traumatic perturbation of the central canal, as histological examination revealed the central canal was occasionally damaged by the injection needle penetrating too deeply through the dorsal columns (not shown). Consistent with this, additional mice were injected with LPC or PBS into the DLF white matter, thereby avoiding central canal damage, and these showed no increase in ependymal zone proliferation (not shown). This finding, when combined with the observation that the remyelination process is well advanced or even completed at 35 days post-LPC treatment, suggests that the proliferation of EpCs is not critical for remyelination in this particular model.

Mice in the EAE model of autoimmune-induced multi-focal demyelination also did not show any increase in numbers of ependymal Ki67+ cells (Figs. 4–5), despite displaying widespread increases in cell proliferation across the spinal cord (Fig. 2E), decreases in LFB-stained white matter (Fig. 1F), and significant functional deficits in locomotor behavior (Fig. 1F).

Quantification and summary comparison of changes in Ki67+ ependymal zone cells in the SCI, LPC and EAE models is shown in Figure 5. Following SCI, the injured Medial segment exhibited a peak in EpC proliferation at 3 dpi at approximately 33 cells/section, in the Rostral segment at 14 dpi at 27 cells/section, and the Cervical segment at 21 dpi at 7 cells/section. No increase was detected in the Caudal segment, and interestingly, a slight depression in proliferation was observed in both the Medial and Caudal segments at 7 dpi. In contrast to SCI, neither chemically-induced nor autoimmune-mediated demyelination resulted in proliferative activation of central canal EpCs.

## Discussion

EpCs surrounding the central canal of the adult spinal cord express neural stem cell properties when isolated *in vitro* and after SCI *in vivo.* While directing the responses of reactive EpCs is an attractive potential approach for improving the spinal cord’s intrinsic repair mechanisms, neither the extent of EpC proliferation following SCI nor the generality of such proliferative responses in cases of non-traumatic spinal cord pathologies is currently well understood. In the present study, we assessed the spatio-temporal characteristics of EpC proliferative reactions in the IH contusion model of SCI, and then performed a side-by-side comparison of EpC proliferative reactions following SCI, chemically-mediated focal demyelination, and MS-like autoimmune-mediated multifocal demyelination. As discussed below, our data help provide a more complete picture of the extent to which latent EpCs are activated by diverse types of spinal cord pathologies.

### Dynamics of Ependymal Cell Reactions in the IH Contusion Model

The IH contusion model provides an excellent experimental approximation of human SCI, and has been endorsed by the Reeve Foundation and most experts in the field as the gold standard for SCI research. The model recapitulates key aspects of human SCI-associated pathology, including inflammation, blood-spinal cord barrier disruption, neuronal and glial cell death, glial scarring, and myelin loss [26]–[28], [31], [32]. Importantly, this device generates remarkably similar lesions between animals, and the intensity of the lesions (i.e. impact force) correlates well with lesion volumes and observed motor and behavioral deficits [27], [28], [31].

Three main conclusions can be made concerning the spatio-temporal kinetics of EpC reactions to the IH contusion model. First, EpC proliferation is rapidly induced in the immediate vicinity of the lesion, and the peak of this proliferation extends away from the injury site in a time-dependent manner. Second, the magnitude of this wave of ependymal proliferation diminishes at greater distances from the lesion, but an approximate 5-fold increase in EpC proliferation still occurs at the cervical level following a low thoracic (T9–10) injury. Third, ependymal proliferation outside of the site of injury is asymmetric, peaking at 3 days within the lesion block, 14 days in an adjacent rostral tissue block, and 21 days at a more distant cervical location, but is absent in a more caudal direction from the lesion segment.

The observation of long-distance and prolonged EpC proliferation in the IH model supports the concept of targeting reactive EpCs to enhance the spinal cord’s intrinsic repair capacity. While we did not trace the fate of the proliferating EpCs, previous fate-mapping studies have shown that, at least in the vicinity of the lesion site, the vast majority of EpC progeny adopt an astrocytic fate after SCI, with small numbers of oligodendrocytes also being generated [1], [17], [22]. Potential therapeutic goals involving endogenous EpCs include modulating their astrocytic contribution to the glial scar, enhancing their rate of oligodendrogenesis, and stimulating their production of replacement or bridge neurons, as such mechanisms are believed to contribute to the functional benefits occurring following neural stem cell transplantation [33], [34]. Interestingly, although reactive EpCs spontaneously generate astrocytes and oligodendrocytes following SCI *in vivo,* new neurons have never convincingly been shown to be produced within the adult spinal cord. Since cultured EpCs can spontaneously differentiate into all three of these neural cell types *in vitro,* it appears that the post-SCI environment is dominated by pro-proliferative, pro-astrocytic, and anti-neurogenic factors.

### Absence of Ependymal Cell Responses in Non-traumatic Demyelinating Models

We assessed EpC proliferation in two distinct models of non-traumatic demyelinating conditions. LPC injection in mice is characterized by a rapid and focal demyelination at the site of its administration, with a subsequent remyelination that is already well manifested by 35 days. EAE is characterized by an autoimmune reaction directed against myelin proteins (in this case, MOG) that results in multifocal demyelination in the spinal cord and brain. Surprisingly, considering that hundreds of studies have used EAE as an experimental model of MS, the real extent of remyelination occurring spontaneously in MOG-induced chronic EAE in C57BL/6 mice has remained elusive. Despite the possibility that remyelination is scanty or absent in this model, several groups have reported the proliferation of oligodendrocyte progenitors and their differentiation into myelin-producing cells in the damaged CNS of MOG-induced EAE mice [35]–[37]. The combination of both the LPC and EAE mouse models should therefore be able to cover a broad range of biological responses occurring in MS, including demyelination and remyelination.

The main conclusion that can be drawn regarding these demyelination models is that neither model elicits an increase in EpC proliferation. A recent lineage tracing study likewise found no evidence for increased EpC proliferation in EAE [38]. Our negative finding in the LPC and EAE ependyma is despite the fact that both of the models were associated with increased overall proliferation (with distinct spatial and temporal characteristics) within the spinal cord (Fig. 2). The absence of EpC proliferation is further surprising in light of the fact that EpCs represent a major proliferative population in both the normal and traumatically injured spinal cord. The lack of EpC proliferation in these models indicates that neither the demyelination/remyelination process nor the complex immunological responses associated with autoimmunity is sufficient, by itself, to provide a proliferative trigger for EpCs. To the contrary, in EAE, baseline EpC proliferation tended towards a decrease.

It should be noted that the absence of EpC proliferation does not rule out the possibility of non-proliferative EpC recruitment in non-traumatic spinal cord pathology models. In this regard, there is some evidence of enhanced EpC migration in a transgenic mouse model of amyotrophic lateral sclerosis (ALS) in which motoneurons degenerate [39].

### Implications for the Signals Activating Proliferation of Latent Ependymal Cells

The differential proliferation of EpCs following traumatic SCI versus non-traumatic spinal cord demyelination may be linked to the distinct gliogenic responses observed in each case. Following SCI, EpCs primarily produce Sox9^+^ GFAP^−^ astrocyte-like cells [1], [18] that synthesize CNTF, HGF and IGF-1, and recent work from the lab of Jonas Frisén indicates that these cells are an important component of the protective astroglial scar [18]. In EAE, although astrocytic barriers reminiscent of those induced upon SCI have also been described, these occur on a much smaller scale and are restricted to areas in close proximity to blood vessels [40]. The absence of EpC proliferation during EAE suggests that EpCs only contribute minimally to the scar-like perivascular barriers that restrict leukocyte entry and spread during EAE, and that EAE is not a strong inducer of astrogliogenic signals in the central canal microenvironment. In future work, it would therefore be interesting to determine whether Sox9^+^ GFAP^−^ astrocyte-like cells are present in models of EAE and LPC. Rigorous testing of this hypothesis would require the use of an inducible and ependymal-specific transgenic fate-mapping model, enabling tracking of migrating ependymal cell progeny. Unlike the pro-astrogliogenic environment occurring after SCI, the environment found in both the LPC and EAE models involves extensive demyelination and, in the case of LPC, remyelination. Thus, neither demyelination nor remyelination supplies proliferative signals for ependymal cells.

### Perspectives

The greatly differing proliferative responses of EpCs in traumatic SCI versus non-traumatic demyelination models highlights the necessity of deciphering the molecular signals controlling activation of latent EpCs. The physiological sources of such activation signals could be diverse, such as: the distinct immune reactions associated with each model; cellular stress and/or cytotoxicity; and the influx of blood-derived factors and/or cells following disruption of the blood-spinal cord barrier. Future work will focus on identifying the endogenous regulators of EpC reactions to tissue injury, in order to develop the potential of harnessing endogenous EpCs to promote spinal cord repair.
